# Structure of Spontaneous UP and DOWN Transitions Self-Organizing in a Cortical Network Model

**DOI:** 10.1371/journal.pcbi.1000022

**Published:** 2008-03-07

**Authors:** Siu Kang, Katsunori Kitano, Tomoki Fukai

**Affiliations:** 1 Laboratory for Neural Circuit Theory, RIKEN Brain Science Institute, Wako, Japan; 2 Department of Computer Science, Ritsumeikan University, Shiga, Japan; University College London, United Kingdom

## Abstract

Synaptic plasticity is considered to play a crucial role in the experience-dependent self-organization of local cortical networks. In the absence of sensory stimuli, cerebral cortex exhibits spontaneous membrane potential transitions between an UP and a DOWN state. To reveal how cortical networks develop spontaneous activity, or conversely, how spontaneous activity structures cortical networks, we analyze the self-organization of a recurrent network model of excitatory and inhibitory neurons, which is realistic enough to replicate UP–DOWN states, with spike-timing-dependent plasticity (STDP). The individual neurons in the self-organized network exhibit a variety of temporal patterns in the two-state transitions. In addition, the model develops a feed-forward network-like structure that produces a diverse repertoire of precise sequences of the UP state. Our model shows that the self-organized activity well resembles the spontaneous activity of cortical networks if STDP is accompanied by the pruning of weak synapses. These results suggest that the two-state membrane potential transitions play an active role in structuring local cortical circuits.

## Author Summary

Information processing by the brain relies crucially on neuronal circuits. Therefore, clarifying the structure of the brain circuitry is a crucial step towards understanding how the brain processes information. In particular, the cerebral cortex occupies a large portion of the brain in primates and humans, so the organization of local cortical networks is essential for the emergence of higher cognitive functions. However, the complex structure and computations of local cortical networks remain largely unknown. In this study, we investigate the neuronal wiring and activity self-organizing with synaptic plasticity in a model of local cortical networks. Synaptic plasticity describes how synapses between neuron pairs are modified according to activities of the individual pairs. The irregular activity self-organizing in our model surprisingly resembles the spontaneous cortical activity observed during sleep. Moreover, such an autonomous activity contains a diverse repertoire of precisely timed temporal sequences. Whether local cortical networks produce such precise temporal sequences is currently debated in neuroscience. The self-organization of temporal sequences in the sleep-like state suggests that they may play an active role in learning sensory experiences and motor skills, for which sleep is known to be crucial.

## Introduction

Cortical networks show complex dynamics of intrinsic activity when sensory inputs are absent. Whether this spontaneous activity is a mere idling state of the brain or, rather, an internal state that actively engages in brain functions remains unclear. Recent experimental studies have revealed an important characteristic of the intrinsic dynamics of cortical neurons. *In vivo* and *in vitro* cortical pyramidal neurons exhibit spontaneous transitions of the membrane potentials between a depolarizing UP state and a resting DOWN state [1]–[14]. Results of a multi-intracellular recording study showed that the onsets of the UP state spread from a local focus and that activations originating from multiple cortical sites are almost synchronous [15]. Other experiments revealed a repeated activation of neurons in neocortical slices with a diverse repertoire of precisely-timed temporal sequences [16]–[19]. Since blockade of glutermatergic synaptic transmissions eliminated the membrane potential transitions, recurrent synaptic input is considered to be crucial for these transitions.

These results indicate that the spontaneous cortical activity is not merely a collection of irregular neuronal firing, but is organized into the spatiotemporal patterns that possibly reflect the structure of local cortical networks. We may raise several questions regarding this issue. How can recurrent cortical networks maintain the two-state membrane potential transitions? Does the self-organized spontaneous activity exhibit precise temporal sequences? What is the likely structure of the local cortical networks self-organized through the two-state transition? Does the two-state transition exert a significant impact on the network structure?

To address these issues, we constructed a recurrent network model of pyramidal neurons and fast-spiking interneurons with synapses between pyramidal neurons modifiable by spike-timing-dependent plasticity (STDP) [20]. As observed in cortical networks [17], the minority of the model's pyramidal neurons displays autonomous membrane potential transitions in the absence of synaptic input. We show that, driven by the autonomous activity, the network self-organizes repeated epochs of UP-state propagation that exhibit irregular durations and intervals. This irregular network activity well resembles the experimentally-observed cortical activity if we prune weak excitatory synapses. Furthermore, transitions from the DOWN state to the UP state (UP transitions) exhibit precisely-timed sequences in the self-organized network.

Self-organization of spontaneous two-state transitions was studied partially in our previous model [21]. A novel finding in the present paper is a repeated activation of temporal sequences during network UP states, in which UP transitions propagate through the entire network. While such sequences were not remarkable in the previous model consisting of homogeneous neuronal populations, the inhomogeneous intrinsic properties of the present model induce neuron-dependent excitabilities that promote sequential neuronal activation. We demonstrate the role of the self-organized synapses and the nontrivial interactions between the self-organizing process and the two-state transitions in generating the repeated sequences. In addition, the non-homogeneous intrinsic properties create a wide variety of firing patterns, as observed in experiments.

## Results

### Self-Organized Spontaneous Network Activity

Pyramidal neurons were numbered in the ascending order of the excitability, with neuron #512 having the highest excitability. In this model, the neuron-dependent excitability of each neuron was adjusted by the density of H-current. Some arguments favorable to the use of H-current are given in the discussion. In highly excitable (HiE) neurons, H-current is slowly activated in the DOWN state and eventually depolarizes the membrane potential to cause an UP transition. H-current is rapidly inactivated in the UP state, and a hyperpolarizing current in turn grows by slow activation of Ca^2+^-dependent potassium current, so the neurons may return to the DOWN state. Thus, the HiE neurons display autonomous two-state transitions through the intrinsic mechanism even without synaptic inputs. In the present study, the excitatory neurons with indices >400 were classified as the HiE neurons, which in reality may correspond to the layer 5 pyramidal neurons that initiate the spread of UP state [3].

**Figure 1:**
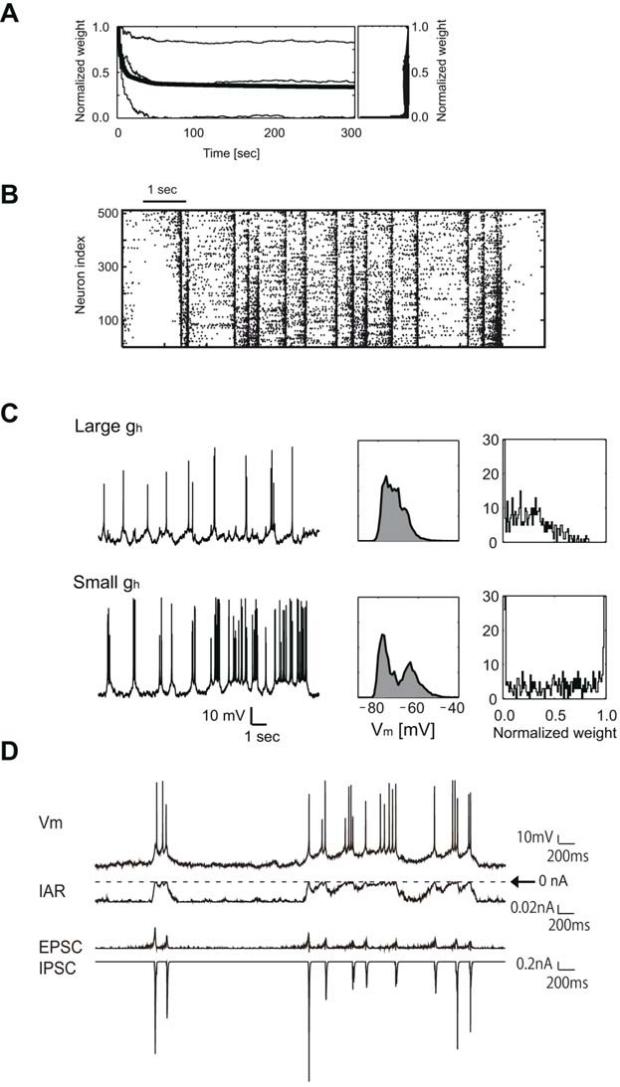
Neuronal activities in the self-organized recurrent network. (A) The time course of normalized weights of recurrent AMPA synapses (*left*). The thick curve shows the value averaged over all synapses and thin curves show three representative synapses. The steady state distribution of the normalized synaptic weights has a bimodal shape (*right*). (B) The network repeated epochs in which pyramidal neurons are sequentially activated to the UP state. Each dot represents an UP transition, and neurons are arranged in an increasing order of maximum conductance of H-current. (C) The model pyramidal neurons displayed a variety of activation patterns depending on the neuron's excitability. The activation patterns of *I*_h_-rich, highly excitable (HiE) neurons were regular, while those of *I*_h_-poor neurons with low excitability (LoE) exhibited spike bursts and were highly irregular (*left column*). Accordingly, the distributions of the membrane potential (*middle column*) and the weights of incoming synapses (*right column*) show different profiles in the two categories of neurons. (D) Time courses of membrane potential, inward rectifier potassium current *I*_AR_, total EPSC and total IPSC in a pyramidal neuron model are shown. A large IPSC hyperpolarizes the membrane potential and in turn activates *I*_AR_ to reset the neuron to the DOWN state. Dashed line indicates the baseline level of *I*_AR_, with inward current shown below the baseline.

Self-organization of recurrent synapses proceeded as in our previous network models with [21] and without [22] two-state membrane potential transitions. The pyramidal-to-pyramidal connections were initially all-to-all and had equal weights, i.e., the half maximum conductance. Due to the activity regulation by STDP, the average weight was reduced to approximately half of the initial value (Figure 1A, *left*), and the weights of self-organized recurrent synapses developed a bimodal distribution (Figure 1A, *right*) [23],[24]. The average weight and bimodal profile remained unchanged once the network reached a stationary state. The self-organized synapses exhibited relatively weak competition in the recurrent network, with the synaptic weights continuously distributed from the minimum to the maximum [21],[22]. Reverberating synaptic inputs induce input-output correlations in individual neurons, and presumably contribute to the strengthening of the weak synapses.

Spontaneous activation of HiE neurons propagated synaptically to the other neurons with low excitability (LoE neurons). Driven by recurrent synapses, activities of the neurons were strongly synchronized in the beginning of self-organization. During the initial stage transient, short-term depression at the pyramidal-to-pyramidal AMPA synapses prevented too rapid increases in firing rate of each neuron, thus preventing too rapid decreases in the maximum synaptic conductance by STDP. Thus, short-term depression made it much easier to maintain the persistent network activity. As recurrent synapses settled down on the steady distribution, pyramidal neurons started to exhibit spatiotemporal activity patterns representing the spread of UP transitions to the entire network (network UP state) (Figure 1B). Recurrent excitation maintained the network UP state, until the negative feedback effects by the activation of Ca^2+^-dependent potassium currents, inhibitory interneurons and synaptic depression would terminate it.

Effects of the various inhibitory feedbacks on self-organization were quantitatively studied in Figure S1A. In general, a weaker inhibition results in higher firing rates of excitatory neurons during network UP states. Since the synapses are significantly modified by STDP during these states, the average synaptic weight in the steady state was decreased (as STDP is LTD-dominant). However, an overly weak inhibition failed to produce epochs of the DOWN state in the spontaneous network activity (see the slowly decaying curves in some of the leftmost panels). Such a continuous UP state made the synaptic weights too weak to maintain the persistent network activity (asterisks in the rightmost panels). Figure S1B schematically summarizes the resultant patterns of network activity.

### Self-Organization with Different STDP Rules

If the network activity could survive the initial-stage down regulation by LTD, a steady state with a moderate firing rate was robustly obtained for additive STDP [23] with different timing windows or different LTD/LTP area ratios (Figure S1C). In contrast, the steady state with a low firing rate was not achieved by other rules of STDP that do not exhibit activity regulation, e.g., a multiplicative STDP rule [25] (Figure S1D). In such a case, all neurons in the network displayed very high-frequency (>150 Hz) tonic firings, in which searching for a structure in network activity is meaningless. The result is also consistent with that of a recent study of self-organization without the two-state transitions using a variant of multiplicative rule [26].

### Activation Patterns of the Individual Neurons

The model neurons displayed a broad range of firing patterns depending on their temporal positions in the activity spread. The HiE neurons exhibited brief UP states with a few spikes, whereas the LoE neurons displayed long-lasting UP states with bursts of spikes (Figure 1C, *leftmost column*). The different activity patterns resulted in quite different profiles of the bi-modal membrane-potential distributions (Figure 1C, *middle column*). The distributions exhibited clearly distinct bimodal peaks in the LoE neurons, whereas such peaks were obscure in the HiE neurons. These results are consistent with the experimentally observed variation of membrane potential distributions [1]–[5],[14],[16]. We note that the distributions of the synaptic weights show quite different profiles in the two neuron types (Figure 1C, *rightmost column*). In HiE neurons, the distribution was strongly biased towards weak synapses due to the asymmetric flow of neuronal activity from HiE to LoE neurons.

Why do pyramidal neurons display this variation of firing patterns? In particular, why do LoE neurons exhibit highly irregular firing patterns when they are driven by the near-regular firing of HiE neurons? In our model, the complex interactions between recurrent inhibition and the inward-rectifier K^+^ current *I*_AR_ enhance the irregular firing of LoE neurons (Figure 1D). A LoE neuron may exhibit UP transitions when it receives volleys of excitatory synaptic input. However, such input does not necessarily elicit a prolonged spike generation. A strong excitatory input is often followed by a strong inhibitory synaptic input, which may quickly hyperpolarize the membrane potential to activate *I*_AR_. Then, the neuron may briefly stop firing, or even make a DOWN transition. Thus, the inhibitory regulation, at least partly, causes irregular firing of the driven neurons.

### Structure of the Self-Organized Neuronal Circuit

**Figure 2:**
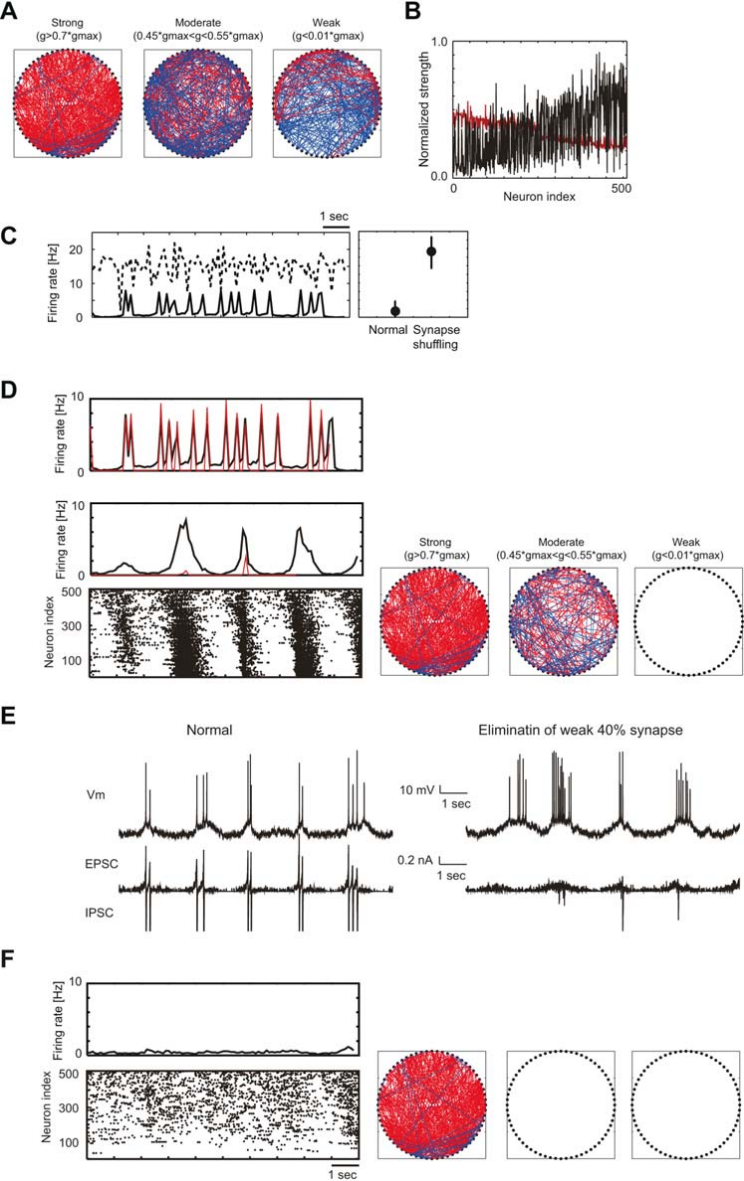
Structure of the self-organized excitatory neuronal network. (A) We selected every 16 neurons from LoE to HiE neurons and arranged the 64 selected neurons along a ring anticlockwise in the ascending order of the excitability. The least excitable neuron is located in the direction of three o'clock. Red lines represent synaptic connections in the feed-forward direction (HiE→LoE), while blue lines show connections in the feedback direction. The left, middle and right panels display strong (g>0.7 g_max_), modest (0.45 g_max_<g<0.55 g_max_) and weak (g<0.01 g_max_) synapses, respectively. Initially, all synapses were set at g = 0.5 g_max_. (B) The average weight of the outgoing (black) or the incoming (red) synapses of the individual neurons. (C) *Left*, The average firing rate of excitatory neurons was simulated with the self-organized synapses (solid) or randomly shuffled ones (dashed). *Right*, The mean and variance of the population firing rate are presented in both cases. The error bars represent SD. (D) *Left*, A network in which the weakest 40% (*g*≲0.4*g*_max_) of all AMPA synapses were eliminated exhibited an irregular activity pattern (*bottom*). We show the average firing rates of the excitatory (black) and inhibitory (red) neuron pools (*middle*). For comparison, we display similar traces obtained in the original model (*top*). *Right*, The ring diagrams represent the resultant patterns of neuronal wiring. (E) Behavior of the membrane potential and postsynaptic current of a pyramidal neuron in the normal (*left*) and 40%-eliminated conditions (*right*). (F) Similar results are shown for the case in which the weakest 60% of the excitatory synapses were eliminated.

To get an insight into the network dynamics, we show the structure of the self-organized neuronal wiring in Figure 2A. Here, we selected every 8 neurons from LoE to HiE neurons (i.e., 64 neurons in total) and arranged them anti-clockwise along a ring in the ascending order of the excitability, with the least excitable neuron at the three o'clock position. Hereafter, we call the information flow directed from HiE to LoE neurons “feed-forward”. Strong, modest and weak synaptic connections are separately shown, and red or blue lines indicate feed-forward or feedback connections, respectively. The figures show that the neuronal wiring is highly asymmetric, i.e., most of the strongest projections are feed-forward whereas almost all weak synapses are feedback. However, some feedback projections are also strong, and they are dense especially among HiE neurons. The activities of these neurons were modulated by the slow intrinsic rhythms, so the relative times of their firing were varied in repeated network UP states. This is why STDP does not eliminate the feedback connections among them. Nevertheless, the feed-forward–dominant organization of the self-organized network is apparent from the average weights of the synapses terminated on or sent from the individual neurons (Figure 2B).

To see whether STDP regulates the activity of recurrent network, we shuffled all the self-organized synapses across the entire population of excitatory neurons. This manipulation keeps the distribution of synaptic weights unchanged over the whole network. However, it mixes up the synapses among the neurons and changes the weight distribution on each neuron. As a result, the synaptic mechanism to regulate neuronal activity was destroyed and the firing rate of excitatory neurons were significantly increased (Figure 2C). Moreover, it eliminated the hyperpolarizing DOWN state (hence, the spontaneous two-state transitions) from the network activity. These results indicate that the additive STDP regulates the activity of the recurrent network, and that such a regulation is crucial for the spontaneous membrane potential fluctuations.

The propagation of neuronal activity in the self-organized network may resemble that observed in cortical networks *in vivo* [27]. However, synchronized network UP states in the self-organized activity occurred in narrower time windows and in more regular temporal patterns than those in the cortical activity. These discrepancies were robustly seen in the spontaneous two-state transitions obtained in our simulations. Interestingly, however, eliminating weak excitatory synapses makes the self-organized network activity better resemble the *in vivo* cortical activity. In fact, the elimination of the weakest 40% of excitatory synapses, which involved a large fraction of the feedback connections (Figure 2D, *right*), generated irregular activity patterns quite similar to the experimentally observed ones (Figure 2D, *left*). Such an elimination of the recurrent synapses significantly reduced the frequency and amplitude of inhibitory feedback synaptic current (Figure 2E), and allowed each neuron to stay in the UP state for longer periods of time. However, a further elimination of the synapses (60% elimination) spoiled the propagation of UP states to the far downstream neurons (Figure 2F). An optimal degree of the elimination exists.

### Precise Temporal Sequences Associated with UP Transitions

**Figure 3:**
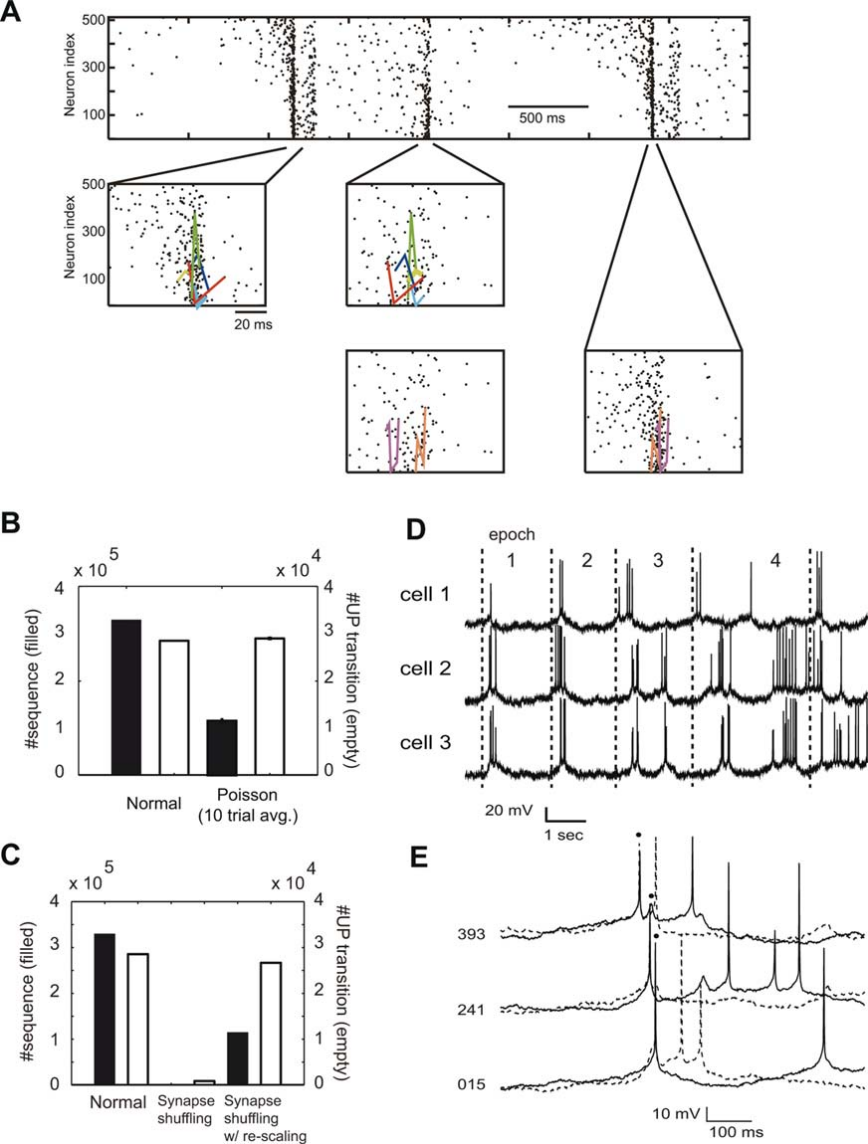
Precise sequences of UP transitions. (A) Each network UP state contained precisely-timed sequences of UP transitions. In the second and third rows, we presented only those sequences that were repeated in successive network UP states. Each sequence allowed timing jitters less than 2 ms, and should be repeated at least three times in a 10 sec-long simulation trial. (B) To test the statistical significance of the sequence generation, we compared the observed number of precise sequences with that of artificial non-stationary Poisson sequences. Both cases generated approximately the same number of UP transitions. In B and C, filled bars refer to the number of UP transitions, while empty bars to that of temporal sequences. Error bars represent SD. (C) A random shuffle of excitatory synapses eliminated most of the UP transitions and temporal sequences (*center*). Rescaling the synapses revived the UP transitions, but not the temporal sequences (*right*). (D) Activities of three excitatory neurons are presented to show the large jitters in the relative times of neuronal firing. For instance, the three neurons fired almost synchronously in epoch 1, while the firing of cell 2 preceded that of the others by about 50 ms in epoch 2. (E) A typical example of the repeated sequences (*solid and dashed traces*), which consist of three neurons, displays different fluctuating patterns of the subthreshold membrane potentials. Dots indicate the UP transitions constituting the two sequences.

We have shown that the self-organized network has a primarily feed-forward neuronal wiring. We tested whether this near–feed-forward structure generates temporal sequences of UP transitions, since such sequence has been reported in cortical networks [16]. To this end, we detected the onset times of the UP state in each neuron by monitoring transient changes in the calcium concentration (see Materials and Methods). By using a template matching method (timing jitters<1 ms: see Materials and Methods), we looked for such precise sequences that consisted of more than two UP transitions and repeated in more than one UP-state epoch. In Figure 3A, we depicted examples of those sequences that were repeated in successive network UP states. The relative temporal relationships between different sequences (e.g., the red and green ones) in general changed in the repetition. Nevertheless, the relative timing of UP transitions within each sequence little jittered.

To confirm the statistical significance of the precise sequences, we constructed a set of N independent non-stationary Poisson event sequences (N is the network size). As shown previously, the individual neurons display quite different temporal activation patterns depending on their relative positions in the UP-state propagation. Moreover, the driven LoE neurons participate in sequences more often than the driving HiE neurons (Figure S2A), presumably reflecting the fact that the driven neurons fire in narrower temporal windows in the repeated network UP states (Figure S2B). In constructing the non-stationary Poisson sequences, we attempted to preserve such a sub-structure of network activity, since it may be crucial for generating the repeated sequences. To this end, we divided the excitatory neural population into eight subgroups of equal sizes and calculated the UP-transition rates in each subgroup in successive time windows sufficiently shorter than the typical duration of network UP states. Then, in each subgroup we set the time evolution of the population event rate of the Poisson sequences equal to thus calculated time evolution of UP-transition rate. Thus, the non-stationary Poisson events preserve the spatial and temporal structure of UP transitions in larger scales, while randomizing the fine spatiotemporal structure of the events (Figure S2C). We found that the number of temporal sequences in the network simulations is significantly larger than that expected by chance (p<0.001) (Figure 3B).

Actually, the neuronal wiring self-organizing through STDP underlies the sequence generation in the present recurrent network. To show this, we shuffled the synaptic connections in a completely random manner. This manipulation destroys the weight distributions on the individual neurons, while preserving the distribution of the synaptic weights over the entire network. Shuffling synaptic connections eliminated most of the DOWN states, and therefore greatly reduced both the number of UP transitions and that of recurrent patterns (Figure 3C). In order to rescue the DOWN states, we rescaled all the excitatory synapses by a factor less than unity. This manipulation recovered the number of UP transitions to the original level, but not that of temporal sequences. The results strongly indicate that the specific structure of self-organized neuronal wiring underlies the repeated sequences.

To further elucidate the role of the self-organized synapses, we examined whether a recurrent network might generate temporal sequences without STDP. We connected pyramidal neurons randomly by excitatory synapses of identical strength, and adjusted the synaptic strength such that the network could exhibit spontaneous two-state transitions at a low rate similar to that of the self-organized network activity. The connectivity of the resultant random recurrent excitation was about 20%. The number of precise sequences was significantly smaller in the random network than in the self-organized one (Figure S3). These results confirm that the precise temporal sequences are a consequence of STDP.

Excitatory neurons were activated nearly in a sequential manner in each network UP state. However, the order of activation was not strictly fixed across different epochs of the network UP state, but rather jittered from epoch to epoch (Figure 3D). Moreover, unlike in experiments [16], the sequences did not generally repeat similar temporal patterns of the subthreshold membrane-potential fluctuations (Figure 3E). The results seem to reflect the fact that the present self-organized network is not a purely feed-forward network (Figure 2A), which would generate only small jitters in the activation order and repeat similar fluctuating patterns of the membrane potential (or postsynaptic current). It is noted that the propagation of UP transitions in cortical neurons was shown to exhibit large timing jitters during slow-wave sleep [15].

### Interactive Effects of STDP and UP–DOWN Transitions on Sequence Formation

If the network is not driven by HiE neurons, the depressing effect of STDP would eventually terminate spontaneous neuronal firing during self-organization. Thus, an apparent role of the membrane potential transitions in the self-organizing process is to maintain the spontaneous activity. Do they also play an active role in sequence generation? We studied this intriguing question by testing whether precise temporal sequences can self-organize without clearly-distinct two-state transitions. To reduce the voltage differences between the two membrane-potential states, we lifted the hyperpolarizing membrane potential of the DOWN state by reducing the maximum conductance of the inward rectifier K^+^ current *I*_AR_. Cortical neurons show a similar suppression of the DOWN state typically when animals are awake or in rapid-eye-movement sleep [4]. However, the mechanism of this change remains unknown, and reducing *I*_AR_ is to be regarded as an ad hoc method to suppress the membrane potential transitions.

**Figure 4:**
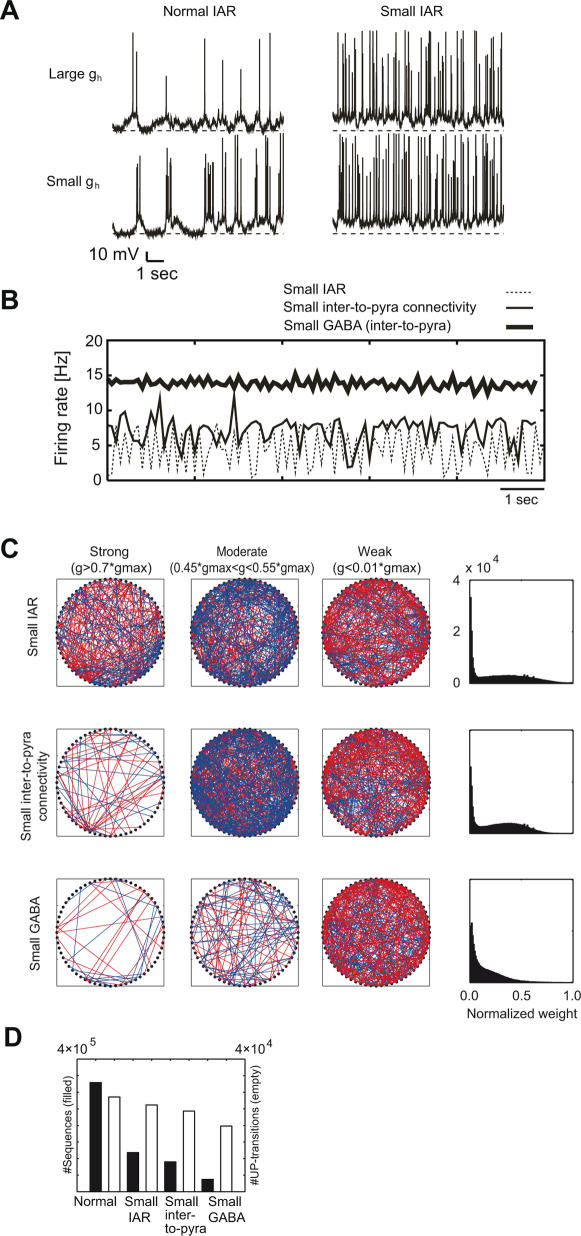
The role of two-state transitions in the network organization. (A) Two cells display typical examples of highly excitable and low excitable neurons (*left columns*). Suppressing the maximum conductance of *I*_AR_ reduces the amplitude of the membrane potential transitions in these cells (*right columns*). (B) The population firing rate of excitatory neurons was calculated in three cases: with a suppressed *I*_AR_; with a reduced connectivity of inhibitory-to-excitatory synapses; with a decreased maximum conductance of these synapses. In all three cases, the membrane potential fluctuations exhibit no clearly distinct DOWN states. (C) The wiring structure (*leftmost and middle two columns*) and the weight distributions (*rightmost columns*) are shown for the synapses self-organized under the above three conditions. (D) The loss of distinct two-state transitions significantly reduces the number of precise temporal sequences in all three cases (*filled bars*), without much reducing that of UP transitions (*empty bars*).

The suppression of the DOWN state changed the membrane potential distributions from bimodal to unimodal (data not shown) and scattered action potentials uniformly over temporal domain (Figure 4A) without much changing the average firing rate (Figure 4B, *dashed*). We then tested whether the recurrent network with the reduced *I*_AR_ could self-organize temporal sequences. STDP failed to develop sufficiently strong synaptic connections, and the feed-forward network structure was obscure compared with the previous case (Figure 4C, *upper raw*). Consequently, the generation of precise sequences was significantly impaired (Figure 4D).

The reason for this reduction of sequences may be understood as follows. The excitatory neurons are sequentially activated during network UP states according to their different excitabilities. The loss of clearly-distinct DOWN states, which was caused by the reduced *I*_AR_, suppresses the neuron-dependence of excitability and the essential difference between HiE and LoE neurons. Therefore, such a loss eliminates the sequential neuronal activation during network UP states. In addition, clearly-distinct network DOWN states reset network activity to prepare for the sequential activation. Thus, the disappearance of DOWN states prevents the development of a feed-forward–dominant network structure.

**Figure 5:**
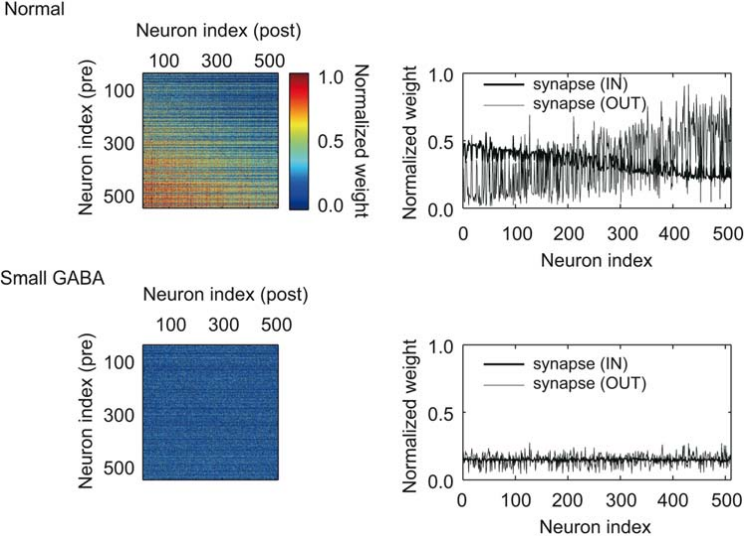
The synaptic matrices obtained in self-organization with the normal and small GABA synapses. Self-organization under the normal condition generated an asymmetric connectivity (*upper left*), while such a structure is not self-organizing under the small-GABA condition (*lower left*). The weights of outgoing and incoming synapses exhibit a systematic neuron-dependence under the normal condition (*upper right*), whereas they show no such a dependence under the small-GABA condition (*lower right*).

We further tested the active role of clearly separated UP and DOWN states by reducing the connection probability or the maximum conductance of the interneuron-to-pyramidal GABAergic synapses. Here, *I*_AR_ was reset to the original magnitude. As in the case of reduced *I*_AR_, the weak inhibitory feedback eliminated, partially or perfectly, the distinct DOWN states (Figure 4B). As a result, the weak inhibition impaired the self-organization of strong synapses (Figure 4C, *middle and lower rows*) and a nontrivial structure of the synaptic matrix (Figure 5). Therefore, the self-organized networks failed to generate precise temporal sequences (Figure 4D).

## Discussion

Computational studies revealed that STDP can self-organize relatively short feed-forward chains to propagate synchronous spikes [22],[28], or networks that produce accurately-timed spike sequences without relying on synchronous firing [29]. We have studied the self-organization of cortical networks while taking the characteristics of spontaneous cortical activity into account, that is, a depolarizing UP and a resting DOWN state. The emergent neuronal network was able to maintain persistent neuronal firing with spontaneous transitions between the two distinct membrane-potential states. We have shown that STDP achieves the balance between recurrent excitation and inhibition to generate the two-state spontaneous transitions. Moreover, the network generated spike sequences with high temporal precision [16]–[17], [29]–[33]. Our results support the hypothesis that cortical information processing may partially rely on accurately timed spike sequences [34]–[40].

### Mechanism of the Two-State Transitions

Blocking AMPA and NMDA synaptic currents terminates the temporally-correlated spontaneous spike sequences in cortical slices [17]. However, layer 5 pyramidal neurons with large apical dendrites exhibit spontaneous regular firing even after these receptors are blocked. In addition, layer 5 neurons initiate the spread of UP transitions in cortical slices [3]. The autonomous activity can be diminished by blocking the persistent sodium current (NaP) and H-current. Since H-current is abundant at the distal sites of apical dendrites in pyramidal neurons [41]–[46], our pyramidal neuron model included these ionic currents in the dendritic compartment. The minority of the model neurons having especially rich concentrations of H-current exhibited self-sustained membrane potential transitions, which contributed to setting the entire network to spontaneous activity (Figure 1B). Since LTD is the dominant component of STDP [47], it rapidly reduces the weights of recurrent synapses during the self-organization. Thus, without the self-sustained activity, the network would easily fall into a permanent quiescent state.

Previous models of UP and DOWN states hypothesized very strong recurrent connections to induce and maintain UP states, and a potassium-dependent intrinsic mechanism [48] or short-term synaptic depression [49] to terminate the UP state. However, due to the powerful down regulation by STDP, recurrent excitatory connections in our model could not remain strong enough to maintain network activity without the intrinsic drive by the HiE neurons. It is likely that under the continuous influence of STDP the persistence of spontaneous activity requires an intrinsic mechanism to initiate the UP state. As mentioned above, here the H-current serves for this role. To turn off the UP state, this model employed a potassium-dependent intrinsic mechanism, short-term synaptic depression and recurrent inhibition (Figure 4B). In principle, we could induce DOWN transitions solely with any one of these mechanisms. In such cases, however, it was difficult to keep the values of the related parameters in moderate ranges. In addition, the network UP states exhibited approximately fixed temporal lengths and regular periodicity (data not shown). The highly irregular repetition of network UP states, such as observed in experiments [27], appeared if the network recruited the multiple mechanisms of DOWN transitions.

H-current is generally considered to be crucial for the integration of synaptic inputs [41],[45]. In addition, some experiments suggested that the blockade of H-current enhances, rather than suppresses, the two-state membrane potential transitions [50]. Whether this current engages in the maintenance of the two-state transitions seems open for future studies. We may replace H-current with some other mechanisms of excitability, such as neuron-dependent resting membrane potentials. In this case, however, the pacemaker activity of the HiE neurons would not be accompanied by slow subthreshold oscillations. Whether the autonomous neuronal firing observed experimentally displays the subthreshold oscillations requires a further experimental clarification.

### Pruning Weak Recurrent Synapses During Self-Organization

We have shown that an additive STDP rule self-organizes such a neuronal wiring that is primarily feed-forward, that is, most synaptic connections are formed to propagate activity from the self-activated HiE neurons to the driven LoE ones. However, the network also develops a non-negligible amount of relatively weak feedback synaptic connections. We have shown that pruning the weak excitatory synapses, most of which are feedback connections, broadens the epochs of network UP state and increases the epoch-to-epoch variability in the duration of network UP states (Figure 2D), as seen in cortical networks [27]. In fact, this prolongation of network UP states is caused by a resultant decrease in recurrent inhibition. These results may suggest that some physiological mechanism eliminates or silences overly weak cortical synapses during self-organization. Note that STDP does not describe such an elimination of synapses. It is intriguing to experimentally test whether such a pruning of cortical synapses follows STDP.

### Roles of Two-State Transitions in Self-Organizing of Recurrent Synapses

Our model predicts that the membrane potential distributions display clear bimodality for the LoE neurons located the downstream of information flow, whereas the bimodality is less obvious in HiE neurons (Figure 1C). The membrane potential transitions in cortical neurons do display a wide variety of temporal profiles [1]–[5]. It seems intriguing to examine whether the experimentally observed pattern of the variations is consistent with our categorization of neurons based on their excitability.

We have shown that the propagation of two-state membrane potential transitions plays an active role for self-organizing precise temporal sequences (Figure 4D). As previously mentioned, HiE neurons provide a powerful synaptic drive on other neurons during self-organization. This synaptic input would activate LoE neurons in the temporal order determined by the resting levels of their DOWN states: the lower the resting potential, the slower the activation of that neuron. The resting level depends significantly on the density of H-current in this model. Then, STDP would strengthen or weaken the synaptic connections between LoE neurons according to the relative order of their activations.

### Comparison with Experimental Observations

Ikegaya et al. attributed the generation of precise temporal sequences to the repetition of the membrane-potential fluctuations that reflect synaptic inputs. However, Mokeichev et al. showed that such repeated membrane-potential fluctuations do not appear more often than the chance level expected from their power spectrum structure, thus rejecting the hypothesis by Ikegaya et al. that the repetition of the membrane-potential fluctuations underlies the precise sequences [51]. In our model, the repeated sequences were rarely accompanied by the repetition of the membrane-potential fluctuations (Figure 3E). This was presumably due to the lack of an obvious feed-forward network structure in our model (note that synaptic connections still play a crucial role in the sequence repetition: see Figure 3C and Figure S3B). Thus, our model suggests that the repetition of the membrane-potential fluctuations is not necessarily required for the generation of sequences. This result seems consistent with that of a statistical analysis by Mokeichev et al. However, our results also imply that the repeated membrane-potential fluctuations do not always provide a good statistical measure for the repeated sequences.

In a broad range of parameter values, the self-organization with UP and DOWN states led the present recurrent network to repetition of network UP states, in which neuronal activations are broadly synchronized (Figure 1). Moreover, pruning the weak synapses made the temporal pattern of the network UP states similar to those observed *in vivo* (Figure 2D, 2E). By contrast, the *in vitro* cortical activity displayed no obvious synchronous activation patterns [16]. Whether the present model may replicate the *in vitro* cortical activity is open for further studies.

*In vivo* cortical neurons typically display the two-state membrane potential fluctuations when subjects exhibit a slow-wave oscillation state [4], [52]–[54]. Boosting the slow oscillations during non-REM sleep improves the ability of declarative memory [55], and the removal of slow-wave sleep significantly disrupts subject's memory [52],[53]. The present results suggest that the two-state transitions may assist local cortical networks in encoding temporal sequences to enhance memory consolidation during sleep.

## Materials and Methods

### Model Neurons

Mathematical details of the model are given in Supporting Information (Text S1). We modified the two-compartment model of pyramidal neurons which was previously introduced for describing the propagation of spontaneous neuronal activity in cortical networks [48]. Thus, the pyramidal neuron is modeled as
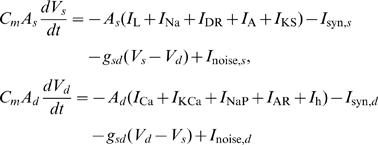
where *C_m_* = 1 mF/cm^2^, *A*_s_ = 0.015 mm^2^, *A*_d_ = 0.035 mm^2^ and *g*_sd_ = 1.75 mS. The somatic compartment involves a voltage-gated sodium current (*I*_Na_), a delayed-rectifier potassium current (*I*_DR_), a leak current (*I*_L_), a transient potassium (*I*_A_), and a slowly inactivating potassium current (*I*_KS_). The dendritic compartment contains a high-threshold calcium (*I*_Ca_), a calcium dependent potassium (*I*_KCa_), a non-inactivating persistent sodium (*I*_NaP_), and a non-inactivating inward rectifier potassium current (*I*_AR_) that is activated at hyperpolarization. In experiments, a minority of pyramidal neurons exhibited autonomous membrane potential transitions even after the blockade of excitatory synaptic transmissions. A hyperpolarization-activated cation current (H current, *I*_h_) and a persistent sodium current have been suggested as the source of this autonomous activity [17]. We included H-current in the dendritic compartment, as it is abundant at the distal dendritic sites of the neocortical and hippocampal CA1 pyramidal neurons [41]–[46]. H-current plays an active role in generating rhythmic firing of thalamocortical relay neurons [56] and globus pallidus neurons [57]. The voltage-dependent kinetics of this current follows those of a thalamocortical relay neuron model [58]. We set the values of parameters and the kinetics of the various ionic currents as given in [48], except for the following: the reversal potential of leak current *E*_L_ = −65 mV; values of the maximum conductance were scaled by 1.2 and 0.5 for an inward-rectifier and a calcium dependent potassium current, respectively; values of the maximum conductance of *I*_h_ were distributed across neurons according to a Gaussian distribution with a mean of 0.004 mS/cm^2^ and a variance of 0.02 (mS/cm^2^)^2^. Cell-dependent strength of the H-current determines different excitability for individual neurons. H-current-rich neurons show spontaneous rhythmic firing at a low rate (<1 Hz) even without synaptic input. All pyramidal neurons received a weak background input represented by a Gaussian white noise with a diffusion constant of 3.0 mV^2^/ms. Following [21],[22], fast-spiking GABAergic interneurons were modeled as
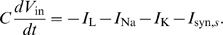



### Network Organization

Our network model has 512 two-compartmental pyramidal neurons and 128 inhibitory interneurons and ∼500,000 synapses including plastic ones for most of the simulations presented here. Pyramidal-to-pyramidal synaptic input is mediated by AMPA and NMDA receptor-mediated glutamatergic synaptic currents, and the pyramidal-to-interneuron synaptic current is mediated by AMPA receptor-mediated synaptic current. Interneuron-to-pyramidal and interneuron-to-interneuron synaptic transmissions, however, are mediated by GABA_A_ receptors. The AMPA and NMDA glutamatergic synapses are located on the dendritic compartments, whereas the GABAergic synapses are located on the somatic components of pyramidal neurons.

The gating variables *s*(*t*) of the AMPA and GABA_A_ synapses are increased by 1.0 at the arrival of a pre-synaptic spike and then decay following a first-order kinetics with a decay time constant of τ_dec_ = 5 ms. The NMDA synaptic current obeys a double exponential function with a raising time constant of τ_raise_ = 10 ms and a decay time constant of τ_dec_ = 50 ms [59]. In addition, the NMDA synaptic current is gated by a voltage-dependent gating variable [59]. Reversal potentials of synaptic currents were set as *E*_AMPA, NMDA_ = 0 mV, *E*_GABA_ = 80 mV, and the values of maximum conductance as 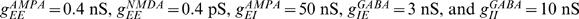


### Synaptic Pasticity Rules

AMPA synapses at excitatory-to-excitatory connections are modifiable by STDP depending on the relative times Δ*t* between an EPSP and a postsynaptic spike [20],[23],[60]:
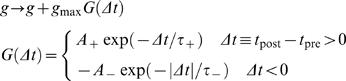
A synapse is strengthened or weakened if the interval from an EPSP to a neighboring postsynaptic action potential is positive or negative, respectively. We employed this additive form of STDP to induce competition between synapses, which has received some experimental support [61]. This rule was chosen to obtain persistent network activity at a moderate firing rate and repeated sequences. However, other study reported a multiplicative STDP rule at cortical synapses [25]. The strength of NMDA synapse between an excitatory neuron pair is rescaled in proportion to that of the AMPA synapse between the neuron pair [62],[63]. The values of the parameters were set as *A*_+_ = 0.005, *A*_−_ = 0.00525, and τ_+_ = τ_−_ = 20 ms. The area law (A_+_τ_+_<A_−_τ_−_) induces a competition between the synapses [23].

The pyramidal-to-pyramidal synapses also exhibit short-term synaptic depression [64]–[66]. The mathematical description of depressing synapses follows that given in [66]. Each synapse is multiplied by a depression factor at every presynaptic spike, while each synapse recovers from the depression in the absence of presynaptic spikes. Depression factors are 0.99 and recovery time constants were distributed over the excitatory neuron population according to Gaussian distributions with a mean of 700 ms and a variation of 50 ms^2^. The connectivity of pyramidal-to-interneuron and interneuron-to-interneuron synaptic connections is 10%, and the interneuron-to-pyramidal synaptic connections have a connectivity of 30%. These types of synaptic connections are not modifiable by STDP. Simulation software was written in C and ran on Pentium 4 3.0 GHz×8CPU PCs using parallel computing by the MPI programming techniques.

Sequence detection.

We marked the UP transitions by the times at which the relative calcium influx defined by Δ[CA^2+^]/([Ca^2+^]+ε) exceeded 2.0 (ε = 0.01 µM). We used a template matching method to detect the precise sequences repeated more than two times. We selected a reference excitatory neuron, say *n*_1_, and picked up the first UP transition in this neuron at time *t*_1_ as a reference event. Then, we searched for all UP transitions that occurred in other neurons after *t*_1_ to construct an event vector, [*t*_1_ *t*_2_ … *n*_1_ *n*_2_ …]. We constructed similar event vectors taking every UP transition in neuron *n*_1_ as the reference event. We compared all possible pairs of thus obtained event vectors as follows. Whenever the same neuron, say *n*_k_, appeared in the two vectors, we examined whether the time differences *t_k_*−*t*_1_ are equal in the two vectors with a precision of ±0.5 ms (criteria 1). If so, we kept the chain *n*_1_−*n*_k_ and repeated the same procedure until we reached the end of the vectors, adding every neuron satisfying criteria 1 to the chain. If the final chain contained more than two neurons (including *n*_1_ itself), we regarded this chain as a precise sequence (criteria 2). We repeated the above procedure for all possible choices of the reference neuron. It is noted that this algorithm might count some sequences in multiple times. However, these redundant counts were negligibly small since the majority of precise sequences contained only three UP transitions in the present simulations (thus, multiple counting was rejected by criteria 2).

## Supporting Information

Figure S1 Parameter dependence of the self-organizing network behavior. (A) The time courses of the population firing rate (left) and normalized average synaptic weight (middle) during the self-organizing process are shown. We conducted the simulations while varying the values of the depressing factor, interneuron-to-pyramidal synaptic connectivity, maximum conductance of the GABAergic synapses and maximum conductance of potassium-dependent calcium current. All these parameters control the overall inhibitory effects on the recurrent network. The average population firing rates are also shown for the steady states of self-organization (right). Error bars show SD. (B) The steady states obtained at various levels of the overall inhibitory effect are schematically illustrated. (C) The self-organizing process was simulated using STDP rules with different timing windows (upper panels) or different LTP/LTD area ratios (lower panels). (D) The equilibrium distribution of synapses obtained by the STDP rule proposed in van Rossum et al., 2000, where LTD obeys a multiplicative rule.
(3.46 MB EPS)

Figure S2 Sub-structure of repeated sequences. (A) The driven LoE neurons (with small indices) have much larger chances to appear in the repeated sequences than the driving HiE neurons (with large indices). (B) The distributions of the times of UP transitions during a network UP state are shown for 100 LoE and 100 HiE neurons (upper). The origin of the time axis indicates the time point at which each network UP state was over. The mean relative times (circle) of Up transitions are shown for the two neuron groups. Error bars indicate SD. The difference in SD is statistically significant (F-test, p<10-7). (C) An example of the non-stationary Poisson event sequences (lower) is shown for the steady state obtained by simulations of the model network.
(0.97 MB EPS)

Figure S3 Generation of UP transition sequences without STDP. (A) The averages firing rates were calculated for a recurrent network, in which excitatory neurons were connected randomly with a connectivity of 10, 20 or 30%. The 20%-connectivity network showed approximately the same firing rate as that of a self-organized network. Error bars represent SD. (B) The number of UP transitions (empty) and that of their sequences (gray) are displayed for the self-organized and 20%-connectivity random networks.
(0.59 MB EPS)

Text S1 The mathematical details of the model given with references.
(0.16 MB DOC)
