# Supplementary material for: Structure of Spontaneous UP and DOWN Transitions Self-Organizing in a Cortical Network Model
Source: PLoS Comput Biol. 2008 Mar 7;4(3):e1000022. doi: 10.1371/journal.pcbi.1000022 (PMC2265465; doi:10.1371/journal.pcbi.1000022)
Supplement: Text S1 — The mathematical details of the model given with references. (0.16 MB DOC) [file pcbi.1000022.s004.doc]

**Supporting Information**

**Details of the Model**

Ionic channels are modeled in the Hodgkin-Huxley formalism, with gating variables x governed by the first-order kinetics equation: $dx/dt=\varphi\left[\alpha_{x}(V)(1-x)-\beta_{x}(V)x\right]=\varphi\left[x_{\infty}(V)-x\right]/\tau_{x}(V)$, where $\varphi$ is the temperature factor.

Pyramidal cells are modeled following [1] with minor modifications. The sodium current, $I_{\text{Na}}=g_{\text{Na}}m_{\infty}^{3}h(V-V_{\text{Na}})$, has a maximum conductance of $g_{\text{Na}}=50\text{mS}/\text{cm}^{2}$, with the activation variable replaced by its steady-state values $m_{\infty}=a_{m}/(a_{m}+\beta_{m})$, where $a_{m}=0.1(V+33)/\left[1-\exp(-(V+33)/10)\right]$ and $\beta_{m}=4\exp(-(V+53.7)/12)$. The inactivation variable has $\alpha_{h}=0.07\exp(-(V+50)/10)$ and $\beta_{h}=1/\left[1+\exp(-(V+20)/10)\right]$. The temperature factor $\varphi$=4.

The delayed rectifier, $I_{\text{DR}}=g_{\text{DR}}n^{4}(V-V_{\text{K}})$, has a maximal conductance of $g_{\text{DR}}=8.4\text{mS}/\text{cm}^{2}$ and the inactivation kinetics are described by $\alpha_{n}=0.01(V+34)/\left[1-\exp(-(V+34)/10)\right]$ and $\beta_{n}=0.125\exp(-(V+44)/25)$, with $\varphi$=4.

The leakage current, $I_{\text{L}}=g_{\text{L}}(V-V_{\text{L}})$, has a constant conductance, $g_{\text{L}}=0.0667\text{mS}/\text{cm}^{2}$.

The fast A-type K^+^ channel, $I_{\text{A}}=g_{\text{A}}m_{\infty}^{3}h(V-V_{\text{K}})$, is taken from [2]. The maximal conductance $g_{\text{A}}=1.0\text{mS}/\text{cm}^{2}$. The fast activation variable is replaced with the steady-state values, $m_{\infty}=1/\left[1+\exp(-(V+50)/20)\right]$, and the inactivation variable is described by $\tau_{h}$=15 ms and $h_{\infty}=1/\left[1+\exp((V+80)/6)\right]$.

The non-inactivating K^+^ channel, $I_{\text{KS}}=g_{\text{KS}}m(V-V_{\text{K}})$, is modeled as in [3], but without an inactivation variable. The maximal conductance $g_{\text{KS}}=0.576\text{mS}/\text{cm}^{2}$ and the activation variable is governed by $\tau_{m}=8/\left[\exp(-(V+55)/30)\right]$ and $m_{\infty}=1/\left[1+\exp(-(V+34)/6.5)\right]$.

The persistent sodium channel, $I_{\text{NaP}}=g_{\text{NaP}}m_{\infty}^{3}(V-V_{\text{NaP}})$, does not have an inactivation variable. The current is modeled following [4] with small modifications. The maximal conductance $g_{\text{NaP}}=0.0686\text{mS}/\text{cm}^{2}$. Since the activation of this channel is fast, the activation variable is replaced with the steady-state values, $m_{\infty}=1/\left[1+\exp(-(V+55.7)/7.7)\right]$.

The inward rectifier K^+^ channel, $I_{\text{AR}}=g_{\text{AR}}h_{\infty}(V-V_{\text{K}})$, is modeled following [5.6], with a maximal conductance $g_{\text{AR}}=0.03084\text{mS}/\text{cm}^{2}$. The current is activated instantaneously below threshold, with a half-activation voltage of -75mV: $h_{\infty}=1/\left[1+\exp((V+75)/4)\right]$.

The high-threshold Ca^2+^ channel $I_{\text{Ca}}=g_{\text{Ca}}m_{\infty}^{2}(V-V_{\text{Ca}})$ is modeled as in [7] with a maximum conductance, $g_{\text{Ca}}=0.43\text{mS}/\text{cm}^{2}$, and the steady state activation variable, $m_{\infty}=1/\left[1+\exp(-(V+20)/9)\right]$. The concentration of intracellular calcium, $[\text{Ca}^{\text{2+}}]$, obeys the first-order kinetics as, $d[\text{Ca}^{\text{2+}}]/dt=-\alpha_{\text{Ca}}A_{\text{d}}I_{\text{Ca}}-[\text{Ca}^{\text{2+}}]/\tau_{\text{Ca}}$ with $\alpha_{\text{Ca}}=0.005\mu\text{M}/(n\text{A}\cdot\text{ms})$ and $\tau_{\text{Ca}}$=150 ms.

The Ca^2+^-dependent K^+^ current $I_{\text{KCa}}=g_{\text{KCa}}[\text{Ca}^{2+}]/([\text{Ca}^{2+}]+\text{K}_{\text{D}})(V-V_{\text{K}})$ is also modeled as in [7] with a maximum conductance, $g_{\text{KCa}}=0.285\text{mS}/\text{cm}^{2}$, and K_D_=30μM.

The hyperpolarization-activated cation channel (H-current), $I_{\text{h}}=g_{\text{h}}m^{2}(V-V_{\text{h}})$, is modeled as in [8] with an activation variable obeying the first-order kinetics. In our model, the values of the maximum conductance $g_{\text{h}}$ are distributed across all pyramidal neurons according to a Gaussian distribution with a mean of $0.006\text{mS}/\text{cm}^{2}$and a variance of $0.02\text{ }\left(\text{mS}/\text{cm}^{2}\right)^{2}$. The activation variable is described by $\tau_{m}=1000/\left[\exp((V+66.4)/9.3)+\exp(-(V+81.6)/13)\right]$ and $m_{\infty}=1/\left[1+\exp((V+69)/7.1)\right]$.

For all the ionic channels, the reversal potentials are given as $V_{\text{L}}=-65\text{mV}$, $V_{\text{Na}}=55\text{mV}$, $V_{\text{K}}=-100\text{mV}$, $V_{\text{Ca}}=120\text{mV}$, and $V_{\text{h}}=-40\text{mV}$.

**References**

1. Compte A, Sanchez-Vives MV, McCormick DA, Wang XJ (2003) Cellular and network mechanisms of slow oscillatory activity (<1 Hz) and wave propagations in a cortical network model. J Neurophysiol. 89: 2707-2725.

2. Golomb D, Amitai Y (1997) Propagating neuronal discharges in neocortical slices: computational and experimental study. J Neurophysiol 78: 1199–1211.

3. Wang XJ (1999) Fast burst firing and short-term synaptic plasticity: a model of neocortical chattering neurons. Neuroscience 89: 347–362.

4. Fleidervish IA, Friedman A, Gutnick MJ (1996) Slow inactivation of Na+ current and slow cumulative spike adaptation in mouse and guinea pig neocortical neurones in slices. J Physiol 493: 83–97.

5. Spain WJ, Schwindt PC, Crill WE (1987) Anomalous rectification in neurons from cat sensorimotor cortex in vitro. J Neurophysiol 57: 1555–1576,.

6. Stern EA, Kincaid AE, Wilson CJ (1997) Spontaneous subthreshold membrane potential fluctuations and action potential variability of rat corticostriatal and striatal neurons in vivo. J Neurophysiol 77: 1697–1715.

7. Wang XJ (1998) Calcium coding and adaptive temporal computation in cortical pyramidal neurons. J Neurophysiol 79: 1549–1566.

8. Wang XJ (1994) Multiple Dynamical Modes of Thalamic Relay Neurons: Rhythmic Bursting and Intermittent Phase-Locking, Neuroscience 59: 21-31.

**Supplementary Figure Legend**

**Figure S1**:

Parameter dependence of the self-organizing network behavior. (A) The time courses of the population firing rate (*left*) and normalized average synaptic weight (*middle*) during the self-organizing process are shown. We conducted the simulations while varying the values of the depressing factor, interneuron-to-pyramidal synaptic connectivity, maximum conductance of the GABAergic synapses and maximum conductance of potassium-dependent calcium current. All these parameters control the overall inhibitory effects on the recurrent network. The average population firing rates also shown for the steady states of self-organization (*right*). Error bars show SD. (B) The steady states obtained at various levels of the overall inhibitory effect are schematically illustrated. (C) The self-organizing process was simulated using STDP rules with different timing windows (*upper panels*) or different LTP/LTD area ratios (*lower panels*). (D) The equilibrium distribution of synapses obtained by the STDP rule proposed in van Rossum et al., 2000, where LTD obeys a multiplicative rule..

**Figure S2:**

(A) The driven LoE neurons (with small indices) have much larger chances to appear in the repeated sequences than the driving HiE neurons (with large indices). (B) The distributions of (B) The distributions of the times of UP transitions during a network UP state are shown for 100 LoE and 100 HiE neurons (*upper*). The origin of the time axis indicates the time point at which each network UP state was over. The mean relative times (circle) of Up transitions are shown for the two neuron groups. Error bars indicate SD. The difference in the mean values is statistically significant (F-test, p<10^-7^). (C) An example of the non-stationary Poisson event sequences (*lower*) is shown for the steady state obtained by simulations of the model network.

**Figure S3:**

Generation of UP transition sequences without STDP. (A) The averages firing rates were calculated for a recurrent network, in which excitatory neurons were connected randomly with a connectivity of 10, 20 or 30 %. The 20 %-connectivity network showed approximately the same firing rate as that of a self-organized network. Error bars represent SD. (B) The number of UP transitions (*empty*) and that of their sequences (*gray*) are displayed for the self-organized and 20 %-connectivity random networks.
